# Hospital Needs

**Published:** 1912-08-31

**Authors:** 


### Hospital Needs.

### ACCIDENT AMBULANCES.

Frequently of late the question has been mooted as to the desirability of hospitals possessing their own ambulances, motor or otherwise, with a view to bringing accident cases to their own doors with dispatch and at less risk to the patients. Accident ambulances are the speciality of Messrs. Wilson and Stockall, of Bury, Lincolnshire, whose designs deserve careful study by hospital managers. The firm in question supplies them for home and colonial use, and are now executing an order for their latest pattern from the Municipality of Bloemfontein.

### A NEW HYPNOTIC.

Pharmaceutical research is now directed on more or less definite lines, and in the search, for instance, for a new hypnotic use is made of the fact that certain relations have been found to exist between chemical formulæ and physiological actions. Of stronger hypnotics there is no need; it is for a milder preparation than any of those newly discovered that there is a vacancy. Soporifics are at the best but mere adjuncts in the treatment of insomnia, and for this reason the preparations which should first be tried are those with a limited intensity of action. A substance fulfilling this object both in theory and in practice as far as can be judged at present is the ester of tertiary amyl alcohol—amylene-hydrate-carbonate—which has been placed on the market under the name of "Aponal." In doses of from 15 to 30 grains this drug acts rapidly and mildly, and is said to be free from all depressant action or undesirable after-effects. Amylene hydrate has somewhat similar properties to paraldehyde and most of its advantages; the combination with carbominic acid produces a solid substance which is of pleasant taste, but is insoluble in water. The London agents for "Aponal" are Messrs. Widenmann, Broicher and Co., of 1 Fenchurch Avenue, E.C., whose preparation "Validol" we have already referred to in this journal.

